# Supplementary material for: Are carbon emissions trading and green financial instruments synergistic? -Comprehensive quantitative research based on content analysis
Source: PLoS One. 2024 Mar 7;19(3):e0298601. doi: 10.1371/journal.pone.0298601 (PMC10919601; doi:10.1371/journal.pone.0298601)
Supplement: S1 Data — (ZIP) [file pone.0298601.s001.zip › Data/地级市Z_F工作报告.zip!/╣π╢1⁄2/╓Θ║ú╩╨/└┤╫╘╣1⁄2╓┌║┼í╛┬φ┐╦╩2╛▌═°í┐_2012.docx]

各位代表：

　　现在，我代表市人民政府，向大会作政府工作报告，请各位代表审议，并请市政协委员和其他列席人员提出意见。

　　一、过去五年工作回顾

　　过去五年，是我市发展进程中不平凡的五年，是抢抓机遇、开创新局的五年，是加速转型、蓄势跨越的五年。面对新的形势和任务，在市委的正确领导下，在市人大及其常委会和市政协的监督支持下，市政府紧密团结带领全市人民，探索科学发展新模式，促进经济社会新发展，完成了“十一五”主要任务，实现了“十二五”良好开局，珠江口西岸核心城市和生态文明新特区、科学发展示范市建设迈出了坚实步伐。

　　经济实力显著提升。经济增速稳中趋快，经济总量不断扩大。全市地区生产总值、工业总产值、财税总收入、地方财政一般预算收入相继跨过1000亿元、3000亿元、400亿元和100亿元大关，预计2011年分别达到1410亿元、3580亿元、459亿元和143.3亿元，年均增长11.3%、11.1%、18.4%和20.4%。人均地区生产总值达1.4万美元，年均增长9.2%，继续位居全省前列。

　　交通枢纽初步形成。交通建设取得历史性突破，珠海正从交通末梢向珠江口西岸交通枢纽转变。港珠澳大桥动工建设；新建市内铁路轨道运输线60.1公里，新增公路69.8公里、桥梁34座，新建高速公路34.5公里；新改扩建港口码头泊位25个，其中5万吨级以上的7个，新增港口吞吐能力4133万吨、货物吞吐量3596万吨，分别增长144%和101%，亿吨大港即将建成。

　　产业升级进程加速。产业朝着高端、集群、错位、生态的发展方向转型，现代产业体系初步形成，三次产业的协调性明显增强。五年累计实现工业增加值2848亿元，比前五年增加1469亿元，年均增长12.5%。规模以上工业企业累计实现利润750.3亿元，比前五年增长194%。预计现代服务业增加值占服务业增加值的比重达55.5%，先进制造业和高技术制造业增加值占规模以上工业增加值的比重分别达42.5%和20.5%。

　　城市格局明显优化。赢得了珠江口西岸核心城市的战略定位，率先探索生态文明发展道路，奠定了发展优势。经济特区范围扩大至全市，横琴新区开发上升为国家战略，城市的区域竞争力和影响力明显提高。调整优化城市功能布局，确定了“一条主轴、两大板块、三区一城、若干组团”的城市格局和组团式紧凑型城市形态。城市化率和建成区绿地率分别提高到87.78%和44%，森林覆盖率达到29%。

　　民生福祉持续改善。民生事业长足发展，社会大局和谐稳定。五年累计用于民生的财政支出达251.9亿元，比前五年增长161.9%。预计2011年城镇居民人均可支配收入达28690元，农村居民人均纯收入达11522元，年均增长分别达10.2%和8.9%。城镇登记失业率连续五年控制在2.8%以内。覆盖城乡的社会保障体系更加完善，在全国率先实施中小学12年免费教育和建立全民医疗保障制度，社区卫生服务机构覆盖率达100%，全市人均期望寿命达80.2岁。

　　五年来，我们围绕建设珠江口西岸核心城市，着重做了以下工作：

　　（一）保增长，壮大综合经济实力。我们紧紧围绕科学发展主题，深入实施《珠江三角洲地区改革发展规划纲要》（以下简称珠三角《规划纲要》），贯彻落实“四年大发展”目标任务，有效应对国际金融危机等重大挑战，采取保增长、保民生、保稳定一揽子政策措施，力促经济持续稳健发展。充分发挥重大项目拉动效应，全力推进十大重点工程建设，五年全市完成固定资产投资总额2254亿元，比前五年增长145%，预计2011年达620亿元，同比增长24.7%，增速位居珠三角第一。外贸转型升级，内需持续扩大，消费日益增长，五年累计外贸出口总额达1026亿美元，比前五年增长119%，加工贸易中委托设计和自主品牌混合生产方式出口所占比重达40%；社会消费品零售总额、旅游业总收入分别达2123亿元和935亿元，比前五年分别增长116%和75%。预计2011年末全市金融机构本外币存贷款余额达2980亿元和1638亿元，分别是2006年末的2.6倍和3.1倍。

　　（二）调结构，提高经济增长质量。我们紧紧围绕转变经济发展方式这条主线，多措并举建设现代产业体系，推动经济发展加速转向内生增长、创新驱动的轨道。

　　集聚发展优势高端产业。制定实施先进装备制造、生物医药、新能源汽车等一系列重点产业发展规划及扶持政策，推动产业高端集聚发展。全市24个工业园（片）区调整为8大特色园区，三灶生物医药等5个产业集群被评为省产业集群升级示范区。做大做强电子信息、家用电气、石油化工等优势支柱产业，全市6大主导产业五年累计完成增加值2209亿元，占全市规模以上工业增加值的79.1%。加快发展高端新型电子信息、生物医药、新能源及新能源汽车等战略性新兴产业，先进制造业实现“上天入海”重大突破，中航通用飞机珠海制造基地、中海油深水海洋工程装备制造基地等重大项目进展顺利，珠海航空产业园成为国家高技术产业基地和国家新型工业化产业示范基地，填补了全省空白，抢占了产业发展制高点。加快发展“两少两有两高”为主的高新技术产业，五年高新技术产品产值累计达5760亿元，比前五年增长139%，占工业总产值的41%。加快发展商务物流、休闲旅游、文化创意、金融服务等现代服务业，十字门中央商务区、横琴长隆国际海洋度假区等重大项目启动建设，成功举办两届中国国际航空航天博览会，银行机构由19家增至26家，服务业和文化产业的增加值五年累计分别达2357亿元和154亿元，年均分别增长10.6%和10.5%。加快发展现代生态农业和海洋经济，斗门生态农业园成为首批国家农业产业化示范基地，海洋经济总产值从247亿元增加到436亿元，增长了76.5%。

　　协调发展内外源型经济。积极扩大增量，带动存量调整优化，推动国资、民资、外资“三足鼎立”。五年累计引进内资326亿元，年均增长58.1%；实际利用外商直接投资59.1亿美元，年均增长6.8%。新引进世界500强企业20家和大型央企项目46个，投资总额分别达3.3亿美元和1911亿元。已建成投产的重大产业项目6个，实现产值35.1亿元；在建项目29个，总投资达840多亿元，建成后年总产值将达2100多亿元。加快发展民营经济，增加值从165亿元增加到340亿元，增长1倍多；扶持中小企业健康发展，通过“四位一体”融资平台累计帮助600余家企业融资45.7亿元，上市公司由13家增至30家。

　　加快建设区域创新体系。实施创新驱动战略，推动出台《珠海经济特区科技创新促进条例》，强化“1+N”自主创新政策的集成效应，不断提升自主创新能力。五年全市财政一般预算安排科技支出累计达21.9亿元，全社会研发投入占地区生产总值的比重由1.3%提高到1.9%。

　　产学研合作不断深化，新增各级公共实验室、企业技术中心和工程中心160家，总数达222家。质量强市建设深入推进，知识产权保护不断加强，在全国率先设立知识产权法庭和检察室。累计专利申请量、授权量比前五年分别增长109%和163%，2011年每百万人口年发明专利申请量居全省第三位，连续六次被评为国家科技进步先进市。

　　（三）兴交通，提升发展带动能力。强化交通的先导性、基础性作用，集中力量推进交通大发展，带动城市格局大转变。加强交通战略规划，规划建设一批总投资达1350多亿元的交通基础设施，其中已动工项目总投资446.3亿元，累计完成投资176.1亿元，完成的投资额是前五年的7.4倍，跨境交通、城市道路、港口码头等基础设施建设取得了重大突破。港珠澳大桥珠澳口岸人工岛陆域形成工程即将完成，广珠城际轨道和广珠铁路全线、高栏港高速和机场高速将于今年先后建成通车。五年累计投资26亿元新改扩建200条市政道路。珠三角首个15万吨级大型干散货码头1号泊位试运行，珠海港成为国家综合运输体系的重要枢纽，江海联运态势形成。空港加快发展，珠海机场五年累计完成旅客吞吐量716.8万人次和货邮吞吐量7万吨，比前五年分别增长99.2%和66.5%。口岸基础设施进一步完善，通关能力大幅提高，五年累计通关人数达4.5亿人次，比前五年增长35.9%。

　　（四）建新区，再创珠海发展优势。国务院批准实施《横琴总体发展规划》，同意横琴实行比经济特区更加特殊的优惠政策，使横琴成为全国开放程度最高、开发政策最优、体制机制最活、创新空间最广的开发新区。推动《珠海经济特区横琴新区条例》制订和颁布实施，为横琴锐意创新、快速崛起提供了法律保障。按照“一年见成效、三年大变化、五年成规模”的开发节奏，高标准创造性地推进横琴各项建设。多联供燃气能源站、横琴总部大厦等一批总投资超过1000亿元的项目正加快推进，15个累计投资超过830亿元的产业项目正抓紧洽谈，路、水、电、气等主要市政基础设施建设取得突破性进展，一期工程将在今年高标准建成。

　　（五）促协调，统筹城市一体发展。落实区域发展总体部署，加快实施主体功能区发展规划，利用特区扩容契机加快城市一体化进程，提高发展的协调性。深入实施“东部大转型、西部大开发”战略，着力打造“香洲服务”和“西部制造”品牌。东部地区产业转型加快，五年累计引进服务业投资205.1亿元，第三产业增加值从727亿元增加到1758亿元。西部地区城市化进程提速，市财政投入50多亿元专项资金加强西部基础设施建设，西部中心城区开发启动；西部地区先进制造业发展成效显著，生产总值、规模以上工业总产值占全市的比重分别从37.8%和50.1%提高到39.2%和53.8%。扎实推进社会主义新农村建设，农村发展水平不断提高。市财政累计投入“三农”资金127.3亿元，年均增长35.1%，累计落实农民生产补贴6.1亿元，促进了农村发展、农业增产和农民增收。新改扩建农村公路87条、里程达117公里，200人以上自然村全部通水泥路，改造危桥21座，改造水利工程及围内水利设施536处，解决了17.2万农村人口饮水安全问题，改善了农村生产生活条件。深入防治地质灾害，加强城乡水利防灾减灾工程建设，创建全国减灾示范区23个。

　　（六）优生态，改善城市人居环境。全面落实“三严”方针，实施生态建设“四个百分百”行动，大力推进生态城市建设，优化了城市环境，增添了城市魅力。

　　加强环境治理。完善环保基础设施，五年累计建成污水处理厂8座，改扩建污水管道250公里，城市生活污水集中处理率、生活垃圾无害化处理率分别从63.6%和64.1%提高到85%和92.3%，城市环境综合整治定量考核连续五年位居全省第一。深入开展“净畅宁美”环境整治行动，市容市貌进一步改善。着力建设“数字城管”，城市管理水平明显提高。

　　实施生态工程。提升绿化水平，全市建成区五年累计新增绿化面积1580万平方米，新增人均拥有城市公园绿地面积3.5平方米，分别比前五年增长34.1%和34.2%。新建扩建公园、小游园20个，建成绿道384公里。创建国家、省、市生态示范区125个、“绿色学校”109所、“绿色社区”34个，全国生态文明试点城市建设卓有成效。

　　节约利用资源。扎实抓好节能减排工作，五年累计单位地区生产总值能耗下降15.03%，化学需氧量、二氧化硫排放量分别下降10.32%和24.26%。推进土地节约集约利用，五年累计清理闲置土地5.3万亩，储备土地2.8万亩，实施“三旧”改造0.6万亩，节地率达20%，每平方公里土地产值和财政收入年均提高11.1%和18.1%。电力、水资源等要素保障能力不断增强。

　　（七）共合作，深化区域交流互动。全面落实粤港、粤澳各项合作协议和优惠措施，不断拓展对外开放和交流合作领域，增强了互利共赢、合作发展的能力。珠港澳台紧密合作深入推进，五年新增港澳台企业1369家，对港澳台进出口总额达505.2亿美元，实际利用港澳台资金33亿美元，年均增长9.7%。粤澳合作中医药科技产业园挂牌运作，总投资9.56亿元的竹银水源工程建成使用，珠澳跨境工业区等合作项目顺利推进，国务院批准珠澳跨境工业区专用口岸增加附属功能。设立全省首个台湾农民创业园，累计引进项目36个，总投资达8亿元。加强珠中江交流合作，协同打造珠中江经济圈，三市签署合作协议44项，实现公交互通、年票互认和通信资费一体化。深入推进“双转移”工作，累计接收本省转移劳动力3.3万人，揭阳、茂名两个对口产业转移园引进项目113个，总投资376.8亿元；已建成投产项目49个，2011年实现产值102亿元。积极做好对口帮扶工作，五年累计投入各类对口帮扶资金6.65亿元，实施扶贫开发“双到”项目495个、实现脱贫9963户；支援汶川绵虒镇灾后重建三年任务两年提前完成，支援重庆三县和四川凉山地区对口帮扶工作扎实有效。

　　（八）惠民生，促进社会和谐稳定。秉承以人为本、执政为民理念，大力保障和改善民生，我市公共服务水平位居全国前列，两次获评“中国最具幸福感城市”，人民群众生活更加幸福。

　　扎实办好民生实事。持续推进惠民生办实事30项工程建设，全市累计完成投资39.3亿元，残疾人综合服务大楼、市一中扩建工程三期、农村体育健身工程等项目顺利完成。扎实办好2011年市政府承诺的十件实事，累计投入15.5亿元，其中镇级福利机构改扩建、社区全科医生培训、公交同城同价等八件实事已基本完成，其他后续工作正加快落实，人民群众得到了更多实惠。

　　提高社会保障水平。实施更加积极的就业政策，就业形势总体平稳，2011年城镇登记失业率比2006年降低0.6个百分点。加快完善社会保障体系，全市社会保险参保人次增长79.2%，月人均养老金增长78.5%，基本医疗保险参保人数突破140万人。城镇职工、农民和被征地农民及城镇居民均实现养老保险和基本医疗保险全覆盖。逐步提高最低生活保障、最低工资等福利标准，累计发放低保金1.3亿元。开展社会救助和扶弱济困活动，累计投入各类扶助资金4.7亿元。加强住房保障体系建设，累计改造华侨农场危房5559套，提供保障性住房19872套（间），发放租赁住房补贴1070万元，解决了10384户家庭住房困难问题，城镇居民人均建筑面积达30.3平方米。

　　发展教卫文体事业。积极推进教育现代化，各级各类教育水平持续提高。五年新改扩建学校94所，至2011年全市新增劳动力人均受教育年限达12.8年。大学园区完成建设目标规模。建立健全公共卫生服务体系，新改扩建市、区、镇三级医疗卫生机构16所，建设“四位一体”农村卫生服务中心131个，医疗卫生服务能力大幅增强，通过国家卫生城市复审。大力开展全国文明城市创建活动，全民文明素质明显提升。加强文化体育工作，规划建设“一院三馆”，不断完善公共文体服务体系，每万人公共文化设施面积达到860平方米，人均拥有体育场地面积达到1.4平方米，均超过全省平均水平。全民健身运动广泛开展，成功举办珠海国际半程马拉松比赛和国际赛车节。

　　维护社会安全稳定。建立健全社会矛盾纠纷排查调处机制，完善四级协调联动的综治信访维稳网络。加强社会治安综合治理，不断提高社会治安立体动态防控能力。全市八类主要犯罪案件发案同比下降44.7%，保持为珠三角地区刑事发案最低、社会治安秩序最好的城市之一。创新安全生产监管模式，构建“一体系三平台”，健全安全生产监管长效机制，安全生产总体形势平稳，2011年事故宗数、伤亡人数分别比2006年下降70%和32%。食品药品、产品质量安全和应急管理工作不断加强，斗门区被评为国家食品安全示范区。

　　统筹发展社会事业。探索建设人口均衡型社会，人口计生工作迈入全国先进行列。加强流动人口服务管理，实行外来务工人员凭积分入户及其子女入读公办学校政策，累计落户1419人、入学3353人。加强双拥共建工作，实现全国“双拥模范城”七连冠和全省“双拥模范城”八连冠。加强司法行政工作，“五五”普法全面完成。志愿者队伍发展壮大，全市注册志愿者人数达13.8万人。推动统计、人防、国防教育、外事、侨务、新闻出版、广播电视、民族宗教、国安、海防、边防、海事、工商、打私、打假、气象、科普、防震减灾、仲裁、档案、地方志、港澳流动渔民、工会、青少年、妇女儿童、老龄、红十字、关工委、老干、慈善和残疾人等事业取得新进步。

　　（九）推改革，增强发展内生活力。坚持先行先试，深入推进“一体两翼”改革。完成大部门制机构改革和第四轮行政审批制度改革，赋予经济功能区依法行使部分市一级行政审批权，市政府工作部门精简1/3，市级审批事项减少58%，审批时限平均减少31.6%，行政许可事项网上办理率居全省第一，政府公共服务公众评价总体满意度连续三年名列全省第一。政务公开工作深入推进，政府网站连续两年获得“中国政府网站领先奖”。加强民主法制建设，自觉接受市人大及其常委会和市政协的监督，累计办结市人大议案、代表建议和政协提案4件、788件和1707件，提请市人大常委会审议地方性法规19项，制定修改市政府规章25项。进一步完善政府系统反腐倡廉惩防体系，促进廉洁从政。突出抓好国有企业改革，市管国有企业由26家重组为13家，国有资本向基础设施、基本公共服务、战略性新兴产业等重点领域集中，国有及国有控股企业资产总额比前五年增加1316亿元，增长189.2%。推行财政资金绩效管理改革，构建财政风险准备金机制，推进部门财政预算公开。全面化解了财政、金融历史遗留风险，妥善解决了政府历史遗留债务。积极推进社会管理创新，政府购买服务、社会组织培育、基层社会治理等改革取得实效，政府购买服务常态化，已向社会组织转移了88项政府职能，政府、社会、公民共建共治共享的社会管理新格局正在形成。

　　各位代表，五年的奋斗征程步履坚实，五年的奋斗成果来之不易，五年的奋斗体会弥足珍贵。实践使我们深刻认识到：

　　一是必须坚持抢抓机遇，增创优势。我们紧紧把握实施珠三角《规划纲要》、开发横琴新区、建设港珠澳大桥为珠海带来的历史性机遇，不断凝聚共识、完善思路、创新举措、破解难题，加速建设珠江口西岸交通枢纽，打造高栏港等先进制造业基地，构建横琴新区等环港澳现代服务业发展高地，赢得了城市转型发展的先机，强化了自身的比较优势，提升了城市战略地位，增强了城市核心竞争力。

　　二是必须坚持与时俱进，改革创新。我们紧跟经济特区未来三十年改革发展、横琴实行更加特殊优惠政策、创新社会管理先行先试和珠海大投入大建设的新形势、新任务，推动特区扩容和法规政策全覆盖，促进横琴区域创新，形成了横琴独具优势的发展环境；深化政府大部门制、国有企业和投融资体制以及社会管理体制改革，形成了加强和创新社会管理的珠海模式，形成了保障新一轮大发展的体制机制，实现了以改革强动力，以改革促发展，以改革保稳定。

　　三是必须坚持统筹互动，协调发展。我们始终坚持发展经济与保护生态并重、“东西互动”与“区域协同”并举、“陆海”开发与城乡建设并进，着力加强生态建设和环境保护，加快“东部大转型、西部大开发”，深化珠港澳、珠中江、珠深穗等区域互动，较好地促进了城乡区域全面协调可持续发展。

　　四是必须坚持民生共进，和谐共建。我们始终贯彻以人为本、执政为民理念，听民声、纳民意、解民忧、纾民困，实施一系列富民惠民工程，切实保障和改善民生，维护社会安宁和谐，人民群众分享到了更多发展成果，更有幸福感。

　　五是必须坚持依法行政，优化服务。我们始终围绕中心、服务大局，加快转变政府职能，坚持科学民主决策，依法履职、强化执行，抓好队伍、转变作风，着力建设效能型机关、服务型政府，营造了务实高效的政务环境，推动了全市经济社会又好又快发展。

　　各位代表，过去五年所取得的成绩，得益于历届市委、市政府打下的坚实基础，有赖于人大、政协和社会各界的鼎力支持，归功于全市人民的群策群力。在此，我代表市人民政府，向全市各行各业辛勤劳动的广大建设者，向人大代表、政协委员、各民主党派、工商联、各人民团体和各界人士，向中央和各省（区、市）驻珠单位、驻珠军警部队官兵，向所有关心和支持珠海发展的港澳同胞、台湾同胞、海外侨胞和国际友人，表示衷心感谢！

　　在总结成绩的同时，我们也清醒地看到，前进道路上仍然面临着许多深层次矛盾和问题。主要是：制约珠海发展的基础设施瓶颈尚未完全打破，城市基础设施特别是交通、能源和市政基础设施仍需加快完善；西部地区城市化水平相对滞后，统筹城市一体化发展仍需加强；现代服务业发展水平还不够高，制造业投资和产业规模还要进一步做大做强，产业结构战略性调整仍处在攻坚阶段；节能减排任务艰巨压力重，资源节约集约利用和生态环境保护力度仍需加大；保障科学发展的体制机制还不够完善，人才支撑能力偏弱，体制机制改革创新和人才队伍建设仍需强化；行政效能还有待进一步提高，行政管理和服务方式仍需优化。对于这些矛盾和问题，我们将高度重视、统筹协调、攻坚克难，加大力度予以解决。

　　二、今后五年总体设想

　　今后五年，是珠海打造核心城市、建设生态文明、推进科学发展的关键时期。纵观当前国内外形势，国际金融危机远没有结束，世界经济增长在短中期内可能呈低迷状态，国际贸易增速回落，国内经济增长放缓，经济运行中的一些突出矛盾没有明显缓解，经济发展的不稳定性、不确定性上升，各类风险交织并存，稳增长、促发展的压力仍然很大。但是，国家宏观调控继续加强，全国、全省经济社会发展持续向好、自主增长的总体态势没有改变，特别是近年来我市交通、产业、城市三大建设实现历史性突破，在探索建设生态文明、走不一样发展道路的进程中，积累了做法经验，创新了体制机制，厚积薄发的比较优势不断强化，重点区域和重大项目的带动效应日益凸显，经济加速发展、内力增强的上扬趋势更为明显，支撑未来发展的根基更加厚实；人民群众对推动我市振兴崛起、建设宜居城市美好生活所凝聚的共识和新的期待，更是我们做好工作的根本保证和力量源泉。总的看来，未来五年有挑战更有机遇，有压力更有动力。我们必须进一步增强机遇意识和忧患意识，顺应形势变化，加强统筹兼顾，提高驾驭能力，稳中求进、好中求快，把握机遇、乘势而为，趋利避害、化解风险，扎实完成好未来五年各项发展目标任务。

　　今后五年，我们要高举中国特色社会主义伟大旗帜，坚持以邓小平理论和“三个代表”重要思想为指导，深入贯彻落实科学发展观，高扬“率先转型升级、建设幸福珠海”主旋律，深入实施珠三角《规划纲要》、《横琴总体发展规划》和《十二五规划纲要》，深入推进“绿色发展、创新驱动、东西互动、区域协同、和谐共享”五大战略，加快转变经济发展方式，推动珠江口西岸核心城市建设取得重大进展，让珠海城市更美丽，让人民生活更美好。

　　我们要顺应人民群众对美好生活的新期待，重点建设交通枢纽、产业基地、宜居城市和人才高地，提升城市功能品质，全面增进民生福祉，着力改善人居环境，把珠海打造成为经济持续发展、环境优美怡人、生活舒适便捷、服务优良健全、文化繁荣昌盛、社会和谐稳定的理想之城、幸福之城。

　　——经济持续发展。以科学发展为主题，以加快转变经济发展方式为主线，推动经济结构战略性调整，大力推进产业基地建设，增强经济综合实力。深入实施创新驱动战略，加快建设人才高地，增强城市发展的支撑力。加快建设法治化、国际化营商环境，增强城市整体竞争力。到2016年，全市地区生产总值超过2800亿元，人均地区生产总值达2.3万美元；规模以上工业增加值超过1300亿元，先进制造业占规模以上工业增加值的比重、现代服务业占服务业增加值的比重、战略性新兴产业占地区生产总值的比重分别超过48%、60%和10%；研发经费支出占地区生产总值的比重超过2.3%。

　　——环境优美怡人。深入实施绿色发展战略，扎实推进生态文明建设，强化规划的龙头作用，完善城市一体化的布局和形态，建设资源节约、环境友好、生态平衡的宜居城市。在全社会树立环保自觉，提高环保责任担当。加强城市环境综合治理，加大生态建设力度，优化提升环境质量。大力美化城市，提升城市魅力。倡导健康文明、低碳环保的生活方式和消费模式，创建低碳发展示范区，加快规划建设低碳城市。深入推进东西互动战略，提升城市一体化发展水平。统筹镇村协调发展，建设生产发展、村容整洁、乡风文明的宜居村庄。到2016年，森林覆盖率超过31%，建成区绿地率达45%，城镇人均公园绿地面积达16平方米，城市空气质量二级以上天数位居全省前列，集中式饮用水水源地水质达标率达100%，城镇生活垃圾无害化处理率达100%，城镇生活污水集中处理率达95%以上。

　　——生活舒适便捷。大力建设交通枢纽，加快构建网络完善、安全高效、便捷通畅的现代综合交通运输体系。积极发展绿色公共交通，加快推进快速公交系统和城市轨道交通的规划建设，构建以公交枢纽为节点、以大运量公交为骨架、以常规公交为主体、以出租汽车等为补充的一流公交体系。加大信息基础设施建设力度，营造城市发展智慧化环境，建设“智慧城市”。进一步完善城市基础设施，形成更为完备的供电、供水、供气、通信、商业、旅游等基础设施网络，缩小生活服务半径。深入实施区域协同战略，提高对外开放和交流合作水平，促进区域协调发展，共建优质生活圈。到2016年，城镇化水平超过90%；公共交通出行分担比率超过30%；全市无线宽带网络覆盖率超过75%，家庭上网平均带宽提高10倍以上。

　　——服务优良健全。建立健全公共财政对民生社会事业投入的稳步增长机制，大幅提高社会福利和公共服务的供给水平，逐步实现基本公共服务均等化，促进社会事业全面发展。到2016年，基本公共服务支出占财政一般预算收入的比重超过30%，城镇居民人均可支配收入和农村居民人均纯收入年均增幅分别超过10%和10.5%，努力实现居民收入增长与经济发展同步；教育现代化水平显著提高，达到同期中等发达国家平均水平，在公办或具有公办属性幼儿园入读的幼儿比例超过30%，学前三年教育毛入园率、高等教育毛入学率分别超过95%和55%；优化15分钟健康圈，率先实现人人享有优质医疗卫生服务目标，居民主要健康指标达到发达国家平均水平；建立社会养老服务体系，最低生活保障和医疗救助实现应保尽保；五年城镇新增就业超过20万人次，城乡三项医疗保险参保率提高到98%以上，城镇基本养老保险参保人数达到100万人，全市城镇保障性住房覆盖面达到20%左右。

　　——文化繁荣昌盛。大力推进文化强市建设，促进文化大发展大繁荣。到2016年，歌剧院、博物馆、规划展览馆、文化馆等一批现代化、标志性的重大文化设施相继建成，国际商务休闲旅游度假区逐步形成。全民健身运动深入开展，城乡基本公共文化服务能力大幅提升，文艺精品不断涌现，群众性文体活动丰富多彩，城市文化特色逐步凸显。人民精神生活更加富裕，城市文明程度、市民文化素质明显提高。文化产业加速发展，文化产业增加值实现翻一番。

　　——社会和谐稳定。深入实施和谐共享战略，加强和创新社会管理，深入推进社会发展服务体系、社会关系协调体系、社会安全防控体系建设，加快形成党委领导下的政府、社会组织、公民共建共治共享的社会管理新局面。突出抓好社会治安综合治理，依法严厉打击各类刑事犯罪活动，强化安全生产、食品药品和产品质量监管，提高突发事件处置能力，打造全国最安全的城市之一。继续加强双拥共建和民兵预备役工作，巩固军政军民团结的大好局面。更加注重提高社会发展的包容性，切实加强人文关怀，统筹兼顾城市各阶层群众尤其是外来人口、弱势群体的发展诉求，实现经济社会发展成果的共建共享，营造兼容并蓄、平等和谐的社会氛围。

　　各位代表，回顾过去，成绩鼓舞信心；展望未来，前景催人奋进。我们要切实肩负起时代和全市人民赋予的重托，努力推动我市经济社会发展实现新跨越！

　　三、2012年工作部署

　　今年是新一届政府履职尽责、实现未来五年目标的起始之年，是全面落实珠三角《规划纲要》“四年大发展”目标任务的决胜之年，也是推动全市经济社会跨越发展的攻坚之年。当前国内外发展环境仍然较为复杂，我们要充分估计面临形势的严峻性，做好应对各种困难和风险的准备，充分利用各种有利条件和积极因素，紧紧围绕转型升级这一核心任务，牢牢把握发展实体经济这一坚实基础，好字当头，好中求快，促进经济发展方式实现质的转变，保持经济平稳快速持续增长。

　　做好今年工作的总体要求是：认真贯彻落实党的十七届六中全会和中央经济工作会议、省委十届十一次全会及市第七次党代会精神，注重深化改革开放，注重推进转型升级，注重建设宜居城市，注重增进民生福祉，推动经济社会跨越发展，以优异的成绩迎接党的十八大胜利召开。

　　今年经济社会发展的主要预期目标是：实现地区生产总值增长11%左右；规模以上工业增加值增长14%；固定资产投资增长20%；外贸进出口总额增长10%；实际利用外商直接投资增长5%，引进内资注册资本金增长15%；财政一般预算收入增长15%；城镇居民年人均可支配收入和农村居民年人均纯收入均增长11%；城镇登记失业率控制在3.2%以内；户籍人口自然增长率控制在10‰以内；完成省下达的节能减排目标任务。

　　为实现上述目标，我们要重点抓好以下工作：

　　（一）着力转型升级，保持经济平稳较快发展。

　　坚持抓项目、扩投资、促消费、稳外贸相结合，继续推进产业结构调整优化，加快建设现代产业体系，促进经济内生增长，壮大经济规模，提升经济质量和效益。

　　一是紧抓重大产业项目建设。做好项目服务，强化项目保障，推进项目建设，掀起新一轮大建设热潮，带动产业发展上规模、上水平。促成碧辟化工PTA三期、中海油精细化工园、低地板电车、拱北口岸地区商业中心改造等重大项目开工建设。协调推进中海油深水海洋工程装备制造基地和南海天然气陆上终端项目、十字门中央商务区一期工程、金山南方总部基地、航空产业园配套工程等重大项目。促进建成中航通用飞机珠海制造基地、佳能珠海迁建项目、路博润润滑油添加剂、华润聚酯、三一海洋重工等重大项目。

　　二是力促高端产业集聚发展。围绕龙头项目发展上下游产业配套，延伸产业链条，推进高端产业集聚发展。加大产业扶持力度，完善园区配套设施，进一步提升高栏化工、香洲办公自动化设备及耗材、三灶生物医药、高新区软件、井岸计算机及通信设备等5个省级产业集群升级示范区发展水平，培育工业总产值超过500亿元的产业集群2-3个，壮大产业实力。突出抓好首批24个战略性新兴产业重点项目的实施工作，打造一批重大示范工程，加快建设航空产业和软件与集成电路设计产业2个省级战略性新兴产业基地，推动战略性新兴产业集聚和规模化发展。支持LED、新能源汽车、太阳能等高端产品在珠海的推广应用，积极发展电子商务和网络购物等消费新业态。建设港口物流园区、临空物流园区和保税物流园区、斗门综合物流基地，发展现代物流业。加快建设国际商务休闲旅游度假区，发展城市观光、历史文化、游艇休闲、绿道健身、滨海温泉、生态农业等特色旅游，继续办好中国国际航空航天博览会及通用航空展、游艇展、国际打印耗材展和国际半程马拉松赛、国际赛车节、沙滩音乐派对等重大活动，提升旅游业和会展业。培育海洋可再生能源技术、海洋食品加工、海洋保健品研发和海水淡化等新型海洋技术产业，加强海洋资源的保护和利用，发展海洋经济。

　　三是充分发挥投资拉动效应。今年市政府计划投资保持增长，新建续建项目93项，其中重大基础设施及民生工程占87.9%。支持金融改革创新，拓展融资渠道，鼓励扩大民间投资。推进国有经济布局结构调整，加强国有企业董事会建设，发挥市属国企投融资作用，保障重点项目资金需求。深化市、区财政体制改革，按照责权利统一的原则，进一步明晰事权，完善适应基本公共服务均等化和主体功能区建设要求的公共财政体系。坚持开源节流，加强税收征管，壮大财税收入，服务项目建设和经济发展。积极开展招商引资选资，优化整合全市招商队伍、招商资源、招商策划和招商活动，充分发挥产业园区招商引资的主体作用，开展现场招商、微博招商、委托招商、以商招商等活动，围绕产业基地、产业集群发展引进大项目、好项目，重点引进战略性、跨国、创新、港澳台等资源，扩大招商成效，增强发展后劲。

　　四是推动内需外贸协调发展。落实国家宏观调控政策，运用综合手段保障经济平稳运行和物价总水平基本稳定。继续扩大内需、刺激消费，组织举办一系列大型珠货展销和文体旅游活动，提升城乡消费和珠货外销规模。培育外贸转型升级基地，发展服务贸易和服务外包，支持外经贸企业抢抓订单、扩大内销，建立自主性国际营销网络。优化进口结构，扩大能源资源、先进技术、关键设备和零部件进口。完善中小企业服务体系，支持民营经济健康发展，推动经济内生增长。

　　（二）着力交通建设，加快完善城市交通网络。

　　坚持交通先行，继续推进交通基础设施建设，优化和完善城市路网，着力发展绿色公共交通，促进城市交通便捷顺畅。

　　一是完善交通基础设施。统筹投入，加强保障，建成、新建和筹备一批交通基础设施项目，增强城市交通承载力。年内建成港珠澳大桥珠澳口岸人工岛填海工程、广珠铁路全线及疏港铁路专用线、高栏港高速和机场高速、珠海大道珠海大桥至金湾互通立交段、广珠西线高速南屏互通立交，开通金凤路一期工程、三台石路延长段的部分路段，缓解香洲主城区南北通行压力。开工建设横琴二桥、港珠澳大桥连接线、金琴高速翠屏段等工程，力争启动珠海大道西线改造和金湾互通立交项目。加快推进省道S365线井岸二桥至中心涌段、省道S272线白蕉高速路口至湖心路口市政二期段、明珠路和港昌路改造等项目建设。抓紧开展金海大桥、珠海市区至珠海机场城轨线、国道105上冲段改造工程、香海路、港珠澳大桥西延线等项目前期工作。

　　二是发展绿色公共交通。科学规划公交服务体系，优化公交线网布局，提高密度和覆盖率。规划建设西部地区公交换乘中心，完善广珠城轨珠海段沿线各类交通的接驳，完成与全省公交“一卡通”对接。加大新能源汽车投放力度，转换400辆绿色公交车，新建若干个加气站。倡导绿色出行方式，建设主城区公共自行车租赁系统，进一步完善慢行交通系统。开展城市轨道交通建设前期研究。

　　三是提高交通管理水平。进一步完善路网结构，规划建设一批停车场和行人过街设施，缓解道路交通供求矛盾。大力推进智能交通建设，优化交通组织，科学精细管控道路，加强交通管制和现场疏导工作，缓解道路施工期间交通拥堵状况。加强车辆安全行驶监管，预防和惩处各类交通违法违规行为，维护道路交通运输安全。

　　（三）着力横琴开发，推动横琴新区发展大变化。

　　坚持抓政策、推项目、促合作，进一步提升横琴开发热度。抓住横琴实行更特殊政策的有利契机，最大程度地发挥横琴开发政策效应，推动总部经济、商务服务、金融服务、文化创意等产业集聚发展，促进全市开发开放。组织实施《珠海经济特区横琴新区条例》，积极配合省出台横琴开发指导意见，加快推进国家批复优惠政策的细化、配套和实施工作。建设横琴口岸综合交通枢纽等基础工程和环岛监管监控设施，争取早日封关运作。完善横琴对外交通配套设施，畅通横琴对外交通联系。推进岛内主要市政基础设施和丽新星艺文创天地等一批重点项目建设，确保环岛东路、长隆国际海洋度假区首期工程等一批项目竣工投产。积极促进金融创新，推进横琴国际金融服务中心合作项目。实施粤港澳合作项目，推进粤澳合作中医药科技产业园建设，配合澳门大学横琴校区等项目按时保质完工。

　　（四）着力东西互动，发挥城市组团功能和作用。

　　坚持规划引导，支持主体功能区发展壮大，促进东西互动协调发展，增强城市带动集聚辐射能力。

　　一是优化城市规划体系。积极探索城市统一规划、统一建设、统一管理的新机制，统筹城市的规划布局。推进国民经济和社会发展规划、城市总体规划和土地利用总体规划“三规融合”，强化城乡规划的基础性、先导性和统筹作用。按照核心城市、宜居城市的要求，完成城市总体规划修改工作，评估和完善现有重要城乡规划，推进城乡规划全覆盖，进一步完善城市规划体系。加强城乡规划实施情况的执法监管，切实维护规划的严肃性。

　　二是推进主体功能区建设。支持香洲区加快发展总部经济和现代服务业，建设区域服务中心。支持高新区科技创新海岸建设，集聚发展新兴产业。推进保税区二期土地开发，促进保税区产业转型升级。深入实施《广东海洋经济综合试验区发展规划》，支持万山区创建国家级海洋综合开发试验区，加快建设海洋强区。加快开发西部中心城区，启动核心区和起步区建设。支持高栏港区加快建设集装箱、煤炭、矿石、天然气以及装备制造业配套码头等重大项目，确保10万吨级航道投入运行，逐步完善多式联运集疏运体系，加快打造世界级船舶和海洋工程装备制造基地、国家级清洁能源和石油化工基地以及珠江口出海门户枢纽港。支持金湾区加快建设通用航空、生物医药、新能源三大产业基地和航空新城。支持斗门区加快建设先进制造业、现代农业和生态旅游基地及宜居新城。

　　三是促进“三农”发展。加强农村交通基础设施建设，年内开工改建46座危桥。完善扶持政策，加大基础设施建设投入，建设斗门生态农业园。依托现有优势农业资源，发展无公害水产、园林花木等种养业，打造农业“专业特色镇”、“特色品牌村”，培育名特优农业品牌，促进农民增收。推进农村环境综合整治，完善农村垃圾、污水处理设施，重点打造一批新农村建设示范村。加大农田水利建设力度，提高围内农田灌溉、排涝工程体系的能力，促进农业增产。推进城乡水利防灾减灾和地质灾害防治工程建设，加快完成斗门北部四小联围、小林联围、乾务赤坎大联围海堤加固达标工程。完善海岛供水、用电、垃圾处理等基础设施，进一步改善海岛生产生活条件，推进海岛综合开发利用。

　　（五）着力绿色发展，提升城市宜居水平。

　　坚持加强生态建设和环境保护，综合开发利用资源，提高发展可持续性，加快建设资源节约、环境友好、生态平衡的宜居城市。

　　一是优化生态环境。实施《珠海市生态文明建设规划》，推进生态文明建设。加强环保能力标准化建设，扎实做好全国环保模范城市复查迎检工作。完善白蕉、富山污水处理厂及区域配套管网设施，推进横琴、万山海岛污水处理设施建设。推进西部地区生活垃圾处理项目，完善东部地区生活垃圾无害化处理设施。保护性开发利用红树林、水松林、近岸滩涂等湿地资源，推进横琴滨海湿地修复工程建设。启动空气可吸入颗粒物（PM2.5）监测，探索和开展空气质量评价工作。加强饮用水水源地保护，完善城市供水基础设施，保障用水安全。整治前山河、黄杨河、白龙河流域和一批黑臭河涌，防治水体污染。推进全市绿道网、公园、绿化设施和生态景观林带建设，提高城市美誉度。

　　二是提升城市品质。深入推进“净畅宁美”行动，积极创建全国文明城市。继续抓好清污、治乱、疏堵、消烟、除尘、降噪、纠违、增绿、添彩各环节工作，巩固环境整治成果。重点加强城市卫生保洁和市容秩序管理，突出整治背街小巷、农贸市场、城乡结合部的环境卫生和乱张贴、乱摆卖、乱停放、乱搭建等行为。加强违法建设的控制和拆除工作，整治和规范户外广告。逐步美化主城区临街老旧建筑，改造一批老旧横街小巷。落实城市管理主体责任，加强联动执法和监督考核，加快“数字城管”建设，提高城市管理精细化、信息化水平。推进未成年人思想道德建设，加强市民公德教育，引导市民自觉养成良好文明习惯。广泛开展文明村镇、文明社区、文明行业、文明单位等创建活动，扎实做好全国城市公共文明指数测评迎检工作。

　　三是强化节能减排。编制实施低碳发展规划，推动低碳城市建设。构建环境公众参与机制，培育环保社团组织，积极倡导绿色低碳的生活方式和消费模式。加大节能技改投入，积极推广节能产品，着力研发和应用低碳技术。鼓励产业废物循环利用，发展循环经济。大力发展清洁能源，规划建设大型海上生态能源基地，加快推进重点输变电工程建设。严格落实主管部门、企业和监管单位责任制，规范企业准入门槛，全面实施重点企业能源审计和减排考核工作。组织结构性减排，强化重点区域、重点项目排污整治。继续加强燃煤电厂脱硫，全面推行脱硝。开辟节能减排新战场，培育节能新商业模式，深入推进工业、建筑、交通、公共机构和农村地区节能工作，开展工业产业绿色化和发展绿色建筑试点。加强节能减排监察执法，严肃查处环境违法行为。

　　四是高效利用土地。建立国土资源管理新格局。强化土地利用规划的引领作用，坚持节约集约用地，加大闲置土地、烂尾楼清理处置和土地储备力度。探索盘活农村集体用地为工业园区配套生活服务设施的新举措，促进工业园区和农村集体经济发展良性互动。建立工业园区节约集约用地评估考核体系，提升园区开发强度和承载能力。继续推进“三旧”改造工作，探索旧村改造与保障房建设相结合的新方法，打造宜居社区范例。加强国土资源执法，严格保护耕地和基本农田，建立健全基本农田保护经济补偿制度。

　　（六）着力深化合作，推进区域协同发展。

　　坚持深化区域交流合作，增强区域发展协调性和互补性，提升区域整体竞争力和带动力。全面落实粤港、粤澳合作框架协议，推进对港澳服务业政策在我市先行先试。积极推动《粤澳协同发展规划》编制工作。完成拱北口岸改扩建工程，推进珠澳跨境工业区专用口岸增加附属功能项目建设，促进便捷通关。提升台湾农民创业园建设水平，扩大珠台交流合作。深入实施珠中江基础设施、产业布局、基本公共服务、城乡规划和环境保护一体化规划，完成《珠中江区域紧密合作规划》和《珠中江城市空间协调发展规划》编制工作，认真落实三地合作协议，扩大合作成果。深化珠港澳、珠中江在文化教育、社会保障、医疗卫生、体育旅游、应急管理、科技人才、资源环境保护等方面的合作交流，共建优质生活圈。扎实做好产业和劳动力“双转移”工作，加快完成扶贫开发“双到”工作任务，抓好市内欠发达村、四川凉山、重庆巫山等对口帮扶工作。

　　（七）着力创新驱动，增强城市发展支撑力。

　　坚持实施创新驱动战略，进一步提高自主创新能力。

　　一是促进科技创新。全面落实《珠海经济特区科技创新促进条例》，进一步优化创新政策环境。支持国家级、省级科技创新公共服务平台建设，进一步满足重点领域的共性技术需求和公共服务需要。推进工业企业技术改造，重点扶持一批具有自主知识产权、主业突出、具有较强竞争力的企业和项目，新增一批企业技术中心、工程中心。深化产学研合作，扩大产学研联盟，组织重大科技项目联合攻关，在高端新型电子信息、生物医药和新能源汽车等领域形成一批具有自主知识产权的重大成果。推动科技创新在现代农业、城市建设、生态环保、安全健康等领域的应用。支持科技型企业创新发展、上市融资。加强知识产权保护，深入推进质量强市建设。

　　二是建设人才高地。全面落实“1+8”人才政策，实行高层次人才工作津贴、创业补贴、住房保障、子女入学、配偶就业、养老保险和特定医疗服务等措施，营造人才发展的良好环境。实施优秀人才评选奖励制度，组织开展珠海杰出人才奖和突出贡献人才奖的评选工作，激发尊重人才、重用人才的示范效应。开辟高层次人才服务绿色通道，加大对创新创业基地及人才的财政支持力度，鼓励高层次人才来我市从事项目研发、技术创新和自主创业。建设高档公租房，改善高层次人才生活条件。推进港澳青年创业园、留学生创业园等引智项目建设，加强珠海高校人才智力资源综合开发。发挥人力资源开发管理服务机构的作用，提升人才服务水平。

　　三是打造“智慧城市”。统筹做好城市信息网络的规划、建设和管理工作，促进网络资源共享和互联互通。加快部署新一代移动通信网络，分区域、按步骤推进无线宽带城市建设。加强信息基础设施建设和信息资源开发利用，扩大生产、流通、服务、社会等领域的信息化应用面，以信息化、智能化助推城市化。推进政务信息资源共享，进一步提高政府信息化水平。

　　（八）着力改善民生，全面发展社会事业。

　　坚持加大民生投入，今年全市财政预算安排近72亿元，同比增长13.6%，继续推进惠民生办实事30项工程建设，切实保障和改善民生，促进社会和谐稳定，提升人民生活幸福指数。

　　一是促进充分就业。深入开展“双百”工程，大力促进创业带动就业，年内城镇登记失业率控制在3.2%以下，城镇新增就业4万人，就业困难人员实现就业1800人。着力解决困难群体就业问题，统筹做好高校毕业生、农村劳动力等群体就业工作。全面完成基层人力资源社会保障公共服务平台建设任务。加强劳动监察执法，构建和谐劳动关系，维护企业和劳动者合法权益。

　　二是健全社会保障。进一步完善社会保障体系，逐步提高社会保险待遇水平，扩大社会保险覆盖面。健全以最低生活保障制度为基础的综合性社会救助体系，加大以扶老、助残、救孤、济困和基本生活、教育、住房、医疗为重点的社会救助力度，实现低保对象和困难群体救助全覆盖。进一步提高低保标准，推进适度普惠型社会养老服务体系建设，加快发展慈善、社会福利、妇女儿童和残疾人事业。

　　三是强化住房保障。加大住房保障工作力度，加快建设南屏“沁园”、金山花园三期、连湾三期等保障房项目，全面完成华侨农场危房改造。年内计划投资3.2亿元，建设保障性住房2800套，新开工1800套，竣工3300套，对低收入住房困难家庭发放住房货币补贴。加强房地产市场监管，促进房地产市场平稳健康发展。

　　四是优先发展教育。促进义务教育高位均衡发展，支持金湾、斗门创建广东省推进教育现代化先进区，建成文园中学新校区，加快建设市十一中，并推动各区新建一批学校，新增义务教育学位2500多个。加快建设斗门区特殊学校、市职教基地，积极创建国家级示范性中职学校。加强幼儿园规范化建设，全面建成11所公办属性标准化镇中心幼儿园，逐步增加具有公办属性的幼儿园数量，提高幼儿入读比例。促进高等教育走内涵式发展道路，提高办学水平。深入推进校安工程建设，抓好校园、校车安全等保障工作。

　　五是繁荣文体事业。今年全市财政安排2.4亿元用于加快发展文化体育事业，同比增长10.2%。加快推进“一院三馆”和镇（街）、村（社区）文化设施建设，实施基层文化设施全覆盖工程，新增公共文化设施面积6000平方米。抓好公共体育设施、组织、活动和服务四大网络建设，完善市、区、镇（街）、村（社区）四级公共体育设施，新增体育场地面积2.6万平方米。打造一批文艺精品，推动公共文体设施和场所向社会免费开放，广泛开展各类群众性文体活动。加快建设珠海南方影视文化产业基地等龙头项目，发展文化旅游、数字传媒、创意设计、文化产品制造业，打造一批知名文化产业品牌。深化文化体制改革，进一步规范文化市场秩序。推进广播电视数字化、网络化建设全覆盖。加强历史人文资源的保护和利用，支持斗门镇创建全国历史文化名镇，启动我市创建全国历史文化名城工作。

　　六是提升医疗服务。加快创建国家健康城市，进一步完善公共医疗卫生服务体系，实现人人享有基本医疗公共卫生服务目标。加快建设市第二中医院、市人民医院北区、市平沙医院（市第五人民医院）、市疾病预防控制中心和市传染病病区，规划建设市第三人民医院。推进三灶、金鼎、井岸、斗门、唐家等5所卫生院升级改造，提升镇（街）、社区医疗卫生机构服务能力。加快推进医疗就诊“一卡通”。完善社区卫生服务机构配套建设和评价考核机制，继续探索公立医院改革。加强医疗卫生监督执法，维护医疗服务市场秩序。

　　七是加强社会管理。深化社会管理体制改革，创新社会组织发展扶持方式，建立社会组织扶持发展资金及管理制度。建立完善政府职能转移制度，制订政府职能转移目录与政府购买服务目录。改革社会组织登记管理制度，对经济类、公益服务类社会组织实行直接登记管理。加快建设社会组织孵化基地，完善社会组织等级评估机制。加强和引导社会组织规范自律，促进社会组织健康发展。深入推进社区民主自治，增强群众自我服务、自我管理能力。推进社区“六位一体”服务平台建设，完善社区服务体系，提高社区服务水平。实施《关于建设人口均衡型社会的意见》，扎实做好人口计生服务工作，推进人口均衡型社会建设。加强综治信访维稳网络建设和社会治安综合治理，完善安全生产“一体系三平台”，开展食品药品安全执法专项行动，强化食品药品监管，健全公共安全预警和应急管理工作机制。组织开展打击欺行霸市、打击制假售假、打击商业贿赂和建设社会信用体系、建设市场监管体系的“三打两建”行动。扎实推进统计、司法行政、双拥、档案、民族、宗教、外事、港澳、台务、海防、打私、人防、科普、气象、防震减灾等各项工作。

　　（九）着力提升效能，打造人民满意的服务型政府。

　　坚持改进行政管理和服务方式，优化政务环境，提升政府公共服务满意度，推进效能型机关、服务型政府建设。

　　一是为民行政。注重深入基层和工作一线开展调查研究，密切联系群众。充分利用“市长信箱”、“政府热线”、官方微博、网络论坛等平台，建立促进民意表达、汇聚民众智慧、解决民生问题的“直通车”制度和机制，畅通和规范群众诉求表达、利益协调、权益保障渠道，实现政府工作与民意的良性互动。深入推进政务公开，完善新闻发言人制度，提高政府运作的透明度，保障人民群众的知情权、参与权、表达权和监督权。

　　二是依法行政。严格按照法定权限和程序行使权力、履行职责，自觉接受人大的监督、政协的民主监督和社会的广泛监督，认真办好人大议案、代表建议和政协提案。修订完善市政府工作规则，优化和规范行政运行机制，提高依法科学民主决策水平。落实政府年度立法计划，提高政府立法质量。建立健全行政执法责任制，促进文明公正执法。深入开展社区普法工作，推进面向公民的普法教育。

　　三是高效行政。加强市、区、镇（街）三级联动行政服务体系建设，深化行政审批制度改革，进一步明确和落实权责分工，精简审批事项，优化审批流程，提高审批效率。加强政府绩效管理，完善政府部门责任白皮书制度，健全部门间的协调配合机制，增强行政执行力。落实行政首长问责制和行政过错责任追究制度，加大行政问责力度。稳妥推进事业单位分类改革。加强政府公务员队伍建设，政府公务员特别是领导干部必须履职尽责，在任务、矛盾、困难、风险和挑战面前，勇于担当、善于担当，敢于面对矛盾、敢于解决问题，反对敷衍塞责、揽功诿过，反对只循陈规、不思新法，确保政令畅通、执行有力，树立政府公信力和权威。建设“敬业实干、规范高效、团结协作”的机关文化，进一步转变工作作风，坚持少开会、开短会、开有成效的会，坚持少发文、发短文、发有质量的文，坚持少讲话、讲短话、讲管用的话，坚持多想事、真干事、干成事，努力营造心无旁骛、干事创业的良好氛围。

　　四是廉洁行政。各级领导班子和领导干部必须以身作则，认真贯彻《中国共产党党员领导干部廉洁从政若干准则》，履行党风廉政建设“一岗双责”要求，加强廉洁自律，推进廉政建设。深化公共资源交易管理体制改革，加强土地使用权出让、建设工程招投标、政府采购、产权交易、资金使用等重点领域和关键环节的反腐倡廉工作。加大从源头上防治腐败的工作力度，充分发挥各方面的监督作用，强化监察审计工作，坚决查处腐败案件。厉行节约，勤俭办事，严格控制行政性经费开支，继续保持公务购车和用车经费、会议经费、公务接待费用、机关出国（境）费用、办公经费预算零增长，降低行政成本。

　　各位代表，我市的发展正处在一个继往开来的重要历史时期，崇高的使命激励着我们，宏伟的目标鼓舞着我们，人民的期望鞭策着我们。让我们更加紧密地团结起来，在市委的正确领导下，凝心聚力，开拓进取，真抓实干，奋发有为，为建设更加美丽的城市、创造更加美好的生活而努力奋斗！
